# Use of grape by-products in aquaculture: New frontiers for a circular economy application

**DOI:** 10.1016/j.heliyon.2024.e27443

**Published:** 2024-02-29

**Authors:** Martina Quagliardi, Emanuela Frapiccini, Mauro Marini, Monica Panfili, Agnese Santanatoglia, Manuella Lesly Kouamo Nguefang, Alessandra Roncarati, Sauro Vittori, Germana Borsetta

**Affiliations:** a School of Biosciences and Veterinary Medicine, University of Camerino, Matelica, 62024, Italy; b National Research Council—Institute of Marine Biological Resources and Biotechnologies (CNR-IRBIM), Ancona, 60125, Italy; c School of Pharmacy, University of Camerino, Camerino, 62032, Italy

**Keywords:** Grape by-products, Antioxidants, Gut health, Aquafeed, Shelf-life

## Abstract

Grape by-products have already been used in cosmetics, food industries, but also animal feed industry, especially monogastrics and in aquaculture. Grape by-products have been studied for a long time and their principal activities are antimicrobial and antioxidant. Concerning aquaculture, the great demand and necessity to replace animal sources with vegetable ones, has placed grape by-products as possible new phytonutrients with beneficial properties. The purpose of this review is to describe the use of grape by-products in aquaculture, during the last decade, concerning their effects on: 1) gut health and welfare status; 2) growth performances; 3) quality of fillets and flesh during the rearing cycle and shelf-life products. Although other studies highlighted that the high supplementation of grape by-products could negatively affect fish health and growth, due to antinutritional factors (tannins), grape by-products are proven to be valuable phytonutrients that can be incorporated into fish feed to enhance growth and health during rearing conditions. Even in fish products, their utilization has proven to elongate the properties and shelf-life of fillets and minces. Further studies to evaluate the possible integrations or replacements with grape by-products in fish feed in order to evaluate their effectiveness in aquaculture from a sustainable circular economy perspective will be desirable to enhance the use of these products.

## Graphical abstract

**Figure:**
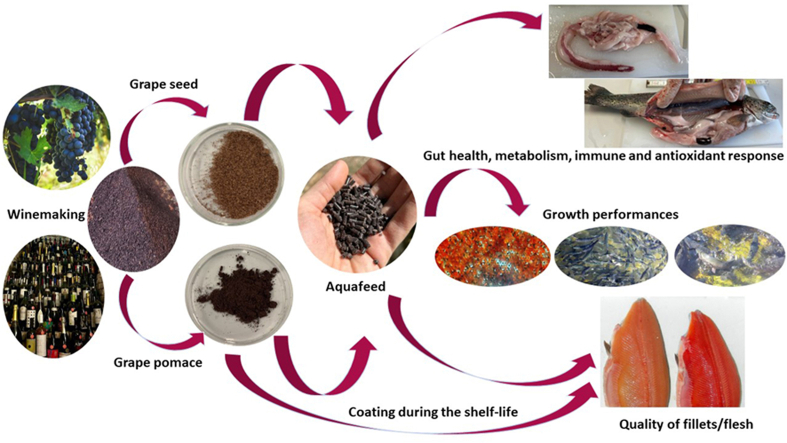

## 1 Introduction

Aquaculture is one of the fastest growing food production sectors in the world, providing nearly 50% of all fish for human consumption. Within the short to medium term (2030), this amount is expected to rise by a further 22% [1]. Aquatic foods provided about 20% of animal protein, reaching over 50% in several countries in Asia and Africa. In the coming years, the growth of aquaculture will therefore require the development of technologies and practices based on a responsible and sustainable approach in line with the strategies of the Blue Transformation, i.e. designing a healthy and environmentally food system where food production and processing are more careful to circular economy [1]. In fact, recently, there has been an increase in interest in the production of self-sufficient fish feed using agri-food wastes and byproducts [2] and the “antibiotic-free” approach [3]. In the last years, aquaculture has been going to adopt codes of prescription to control the problem raised by the antimicrobial resistance and guarantee the quality of the product. Even if aquaculture provides 8% of animal protein intake to the human diet, this industry contributes to rise the antimicrobial resistance. According to a recent survey [4] on the global trends in antimicrobial use, aquaculture carries the highest intensive use per kilogram of biomass; for some aquatic species the antimicrobial application overtakes consumption levels in terrestrial animals and humans. Some organic antibiotic substitutes, like plant secondary chemicals (polyphenols and tannins) have long been utilized in human medicine but have only recently been given particular consideration in animal medicine [5]. Among these compounds, tannins have been shown to avoid bacteria development utilizing a variety of mechanisms, including iron chelation, inhibition of cell wall formation, disruption of the cell membrane, and blockage of the biosynthetic pathways for fatty acids. Tannins have the ability to inhibit the expression of various virulence factors, including biofilms, enzymes, adhesins, motility, and poisons, as well as operate as quorum-sensing inhibitors. Moreover, tannin-loaded hydrogels and nanoparticles exhibit strong antibacterial properties [6].

Some of the bioactive components of grape by-products could be used against foodborne bacteria, such as *Campylobacter jejuni, Escherichia coli, Listeria monocytogenes, Salmonella enterica, Staphylococcus aureus, Vibrio cholerae* and microbial toxins (ochratoxin A, Shiga toxin) in food, because they increase microbiological food safety, prevent or treat animal and human illnesses, and maximize the use of grapes and by-products [7].

Moreover, the wine production sector is one of the most important in the agriculture field at international level. According to the International Organization of Vine and Wine Intergovernmental Organization [8], in 2022 the world wine production was estimated in 258 million hectolitres. Italy (49.8 mhl), France (45.6 mhl) and Spain (35.7 mhl) were at the top of producers’ ranking [8]. These countries are considering the possibility to recovery wastes from wine production from *Vitis vinifera* becoming resources and enhance, through recycling, benefits for both the environment and the economy. A recent paper on the residues from wine production for biorefinery deployment [9] has reported that 130–200 kg of pomace, 30–40 kg of stalks and 15–60 kg of wine lees remain from 1 t of grapevine after processing.

Around 75% of the world grape production is destined to wine industry and the remaining skin, seeds and stalks represent the 25% of total grape weight used in the wine making process. Seedless pomace is about 48–62% of the total grape pomace, source of dietary fibre and phenolic compounds, while the seeds are about the 38–52%, mainly source of oils rich in unsaturated fatty acids [10]. The largest concentration of phenolic compounds, as well as the strongest antioxidant, cytotoxic, and antibacterial activity, were found in the seeds. The skins had the highest concentrations of *p*-coumaric acid hexoside and anthocyanins [11].

For these reasons, grape by-products could be used as dietary supplements as nutraceutical, food or part of this that provides medical or health benefits [12]. Grape by-products are rich in phytonutrients, plant derived compounds and more specifically phytochemicals, bioactive plant-derived compound associated with positive health effects [13]. For example, grape pomace could be used for the production of extracts with antioxidant properties, fermentation substrates, due to its high content of bioactive compounds with high antioxidant activity, such as polyphenols (anthocyanins, flavanols, flavan-3-ols, procyanidins), phenolic acids, resveratrol, and fibre [14].

Phytophenols (phenols of plant origin) can be introduced in aquaculture feeding systems in the raw form (i.e., the whole plant part) rather than in the form of extracts, taking into consideration other possible active ingredients that may be present. Polyphenols, such as resveratrol, a very present stilbene in the red grape *Vitis* vinifera skin, are polyclicic phytophenols and could have an immunomodulatory activity; for example, resveratrol target is the apoptotic cell death [15].

Another natural stilbene naturally present in grapes is the piceatannol, that together with other phenolic compounds such as flavanols, phenolic acids and proanthocyanidins, could reduce the apoptosis induced by the overproduction of reactive oxygen species (ROS) and do a scavenging activity for these reactive species and could activate the nuclear factor erythroid 2–related factor 2 (Nrf2), that regulates the expression of many antioxidant enzymes; here is their fundamental antioxidant activity of these natural compounds. For these reasons, they are considered safe and feasible alternatives to synthetic chemical compounds, positively contributing to the enhancement of fish health status, quality, productivity and food safety, by reducing the use of chemicals and antibiotics in aquatic farming [16].

By the way, during the recent years, other additives have been used as functional feed in aquaculture, such as marine-derived products. In parallel with the phytophenols, these bioactive molecules are involved into the antioxidative and immune responses of fishes. Marine invertebrates are the main source of functional compounds in the marine environment, while plants are the main source in the terrestrial environment. This could be due to the fact that marine organisms are more difficult to gather and identify compared to terrestrial organisms. Polysaccharides extracted from macro and microalgae, crabs, shellfish, corals and fungus, such as alginate, galactan, fucoidan, agar, laminarin, chitin, carrageenan and chitosan, are the most used bioactive compounds in aquafeed [17].

However, numerous studies look into this topic using grape pomace as source of technologically advanced compounds that could be used to improve stability and nutritional qualities in food, pharmaceutical, cosmetic industry, where grape seed oil is widely used and in animal feeding [10].

In livestock, the possibility to use of grape by-products has already been faced out in different studies. A review concerning the use of agro-industrial co-products in animal diets has considered 57 studies on this topic in the last 11 years [18]. It is crucial to determine the proper quantity of supplementation in the animal diet because their chemical compositions highly vary. This is mainly due to the different processing techniques they are subjected to and their seasonal variability and availability [18,19].

Grape by-products have already been used in several animals breeding, testing the qualities they provide [20]. The species more frequently used were dairy cows [[21], [22], [23]], dairy ewes [[24], [25], [26]] horses [27,28] and rabbits [[29], [30], [31]].

By the way, the species in which it has been more widely used are monogastric (pig and poultry), but also unconventional species such as Japanese quail [32,33] and Pekin duck [34]. In addition, to improve the meat's oxidative stability and lower the additives supplementation, like vitamin E, the dietary inclusion of these by-products would also improve the meat's quality. This aim is reached thanks to the direct addition of these natural antioxidants, such as flavonoids, that behave like free radical scavengers and metal chelators, helping to satisfy consumer demand for healthier meat products. This is possible because antioxidants are introduced into the muscle by dietary manipulations through the animal feed or applicated directly in meat and meat products. According to several experts, adding natural antioxidants to animal diets significantly enhances meat quality, when compared to diets without antioxidants, while also slowing down oxidation [35]. Regarding antibacterial activity, they promote the development of some strains of helpful bacteria in the intestinal tract while actively avoiding some dangerous bacteria [36].

A total of 54 papers concerning the effects of grape by-products on pig and poultry growth performances, agree that piglet growth might be improved by the addition of these nutritional sources up to 9% feed, contrarily to poultry, where this outcome was only achieved utilizing by-products up to 3%. Due to the presence of anti-nutritional chemicals, by the high amount of cell wall lignification and polyphenolic chemical content (such as tannin), the inclusion of excessive quantities of grape by-products in chicken diets can hinder growth performance [37]. The same substances can enhance meat quality and nutritional value of pork and poultry meat, ameliorating fatty acid profile (increase of n-3 polyunsaturated fatty acids-PUFA, decrease of n-6/n-3 ratio) and the reduction of susceptibility to lipid oxidation (decrease in thiobarbituric acid reactive substances-TBARS) [38].

However, concerning aquaculture, a review of Dawood et al. (2022) explained the use of fruit processing by-products, among which those derived from grape seed extract, seed oil, micro-incapsulated and pomace meal [39]. The forms in which the derivatives of the grape can be administered are manifold: grape pomace flour, grape seed extract, grape seed oil, microencapsulated. The technologies of extraction and processing, such as enzyme supplementation or pretreatment processes, are very often used in order to improve grape by-products’ bioavailability, because of the high levels of tannins, that are antioxidants but at the same time antinutrients [38,40,41].

The grape by-products could be used in zootechnical feed such as micro-nutrient or macro-nutrient, so the rate of substitution could be different during the rearing phase; on the contrary, they can be used as food additives once the product is ready to be bought, in order to increase the shelf-life [42].

In the aquafeed sector, wine industry by-products had already been explored as source to improve the quality of feed, using their volatile composition to enhance the organoleptic traits and beneficial properties, on the concept of circular economy. Differently, certain acids (like hexanoic acid) and terpenoids (like limonene) might be used as antibacterial, antioxidant, and antiproliferative agents; esters and terpenoids contributed positively to the aquafeeds scent with fruity, sweet, green, fresh, and berry overtones [43].

Based on this context, a review on grape by-products used in aquaculture was purposed with the aim to show the potential application of this by-product in this field, employed not only as micro-macronutrient or additive, but also with their effect on zootechnical parameters and veterinary-pathological characterization. A total of 49 papers of the last ten years of scientific production were taken into account. The antioxidant response of the fishes treated with grape by-products is mainly discussed and proven in every of the articles considered for this review. However, it has been challenging to divide the whole papers considered, because very often they treat multiple transversal topics. Thus, the main outcomings were classified into 3 macro-groups: the effects of grape-by products on fish health (gut, immune system, metabolism); the effects of grape-by products on growth performances; the effects of grape-by products on the quality of fillets and flesh during the rearing cycle and during shelf-life of fillets and fish products.

**1. Gut health, metabolism, immune and antioxidant response of fish fed with grape by-products**

In recent years, the interest of researchers on fish gut health has been increasingly emerging. This especially occurred at a time when fish diets have begun to be replaced with vegetable sources and ingredients alternatives to conventional ones, with the aim of limiting the competition of fish stocks [44]. The most used alternative feedstuffs are soybean meal, which very often caused intestinal inflammation, modulating microbiota and histology [45,46], poultry by-products and insects meals [47,48]. Also for the grape by-products, numerous studies investigated on the effects of their substitution or their use as additives at intestinal level and as consequence both in growth performances and in mitigation soybean meal subacute enteritis-induced [49]**.**

The digestive bioaccessibility of polyphenols has been a crucial first step recognized, also performing *in vitro* studies, because various elements were identified to improve the knowledge of the biological functions of polyphenols for a given species. In general, the gastrointestinal system may function as an extractor, gradually releasing polyphenols from solid matrix and making them available for absorption or enabling the gastrointestinal tract to experience their biological effects [50]. However, a recent study [51], based on *in vitro* technique to determine the potential complexation of wine polyphenols, showed that wine by-products, with either digestive enzymes or feed matrix components, could limit their bioaccessibility. The change of the digestion time was also studied when wine bagasse and wine lees were added to the diets of two fish species: gilthead sea bream (*Sparus aurata*) and flathead grey mullet (*Mugil cephalus*) [51]**.** *In vivo* studies have already been done concerning the effects of phenolic compounds, contained also in grape seed extract and grape pomace, on the intestine, that enhanced the integrity and functionality of the intestinal barrier via three basic mechanisms (Table 1): reduction of pro-inflammatory molecules (tumor necrosis factor α-TNF-α, and other interleukins-IL); improvement in tight-junction protein expression; improvement of the antioxidant intracellular activity [52].

**Table 1:** Effects of grape by-products on gut health, metabolism, immune and antioxidant response of fish fed with grape by-products.

Grape by-product	Most significant dose/(Experiment time, days)	Main findings	Fish species	References
Condensed tannins (CT)	2 g/kg fish feed (63d)	↑intestinal injury, microecological imbalance, metabolic disorder ↓negative effects if combined with PEG (polyethylene glycol)	Chinese seabass (*Lateolabrax maculatus*)	Chen et al. (2022) [53]
	200–400 mg/kg fish feed (56d)	↓trypsin, LPS, *Cetobacterium*, *Aeromonas* ↑*Clostridium, Bravinema* ↔CK, energy metabolism, growth performances	Japanese seabass (*Lateolabrax japonicus*)	Peng et al. (2021a) [54]
	0.5–4 g/kg feed (56d)	↑deformation and histomorphology lesions of intestinal villus ↔nutrient digestibility ↑growth ↑SOD, GPO, Nrf2	Whiteleg shrimp (*Litopenaeus vannamei*)	Peng et al. (2021b) [55]
	1 g/kg fish feed (56d)	↓negative effects of AFB_1_: ↑serum antioxidant capacity and immunity ↓intestinal permeability, alteration of microbiota	Japanese seabass (*Lateolabrax japonicus*)	Peng et al. (2022) [5]
	100–400 mg/kg fish feed (56d)	↓intraperitoneal fat ratio, IL-6 ↑TAOC, CAT ↔growth performance, whole body composition, serum biochemical parameters, fatty acid profile	Japanese seabass (*Lateolabrax japonicus*)	Peng et al. (2020a) [57]
	0.8–1.0 g/kg fish feed (56d)	↓mortality due to the copper sulphate stress ↓intraperitoneal fat ratio, IL-6 ↑liver TAOC, CAT, SOD, glutathione S-transferase, Nrf2 ↔growth performance, whole body composition, serum biochemical parameters, fatty acid profile	Japanese seabass (*Lateolabrax japonicus*)	Peng et al. (2020b) [58]
	20–40 mg/kg fish feed (42d)	↓negative impacts of high soybean inclusion, ↓growth performance, MPO activity ↔morphology and gastrointestinal indices	Yellowtail amberjack (*Seriola lalandi*)	Stone et al. (2018) [49]
Grape seed extract (GSE)	200 mg/kg fish feed (60d)	↑epidermis thickness, goblet cell density, volume density in fish skin ↑intestinal villus height, goblet cell density, intraepithelial lymphocytes ↑C3, Lys, omDB-3, IFN-ɣ, TNF-α genes ↑bactericidal activity *vs Yersinia ruckeri*	Rainbow trout (*Onchorynchus mykiss*)	Mousavi et al. (2021) [59]
	10 g/kg fish feed (60d)	↑glucose, CAT, GPO-1, glutathione S-transferase ↓glucose, MDA	Rainbow trout (*Onchorynchus mykiss*)	Mousavi et al. (2020) [60]
	0.1% fish feed	↓negative effects of oxidized fish oil: ↓hepatocytes hydropic (vacuolar) degeneration ↑HSI, liver biochemistry parameters (SOD, GSH, MDA, ALT, AST, ALP)	Rainbow trout (*Onchorynchus mykiss*)	Terzi et al. (2023) [63]
Grape pomace flour (GPF)	300 mg/kg fish feed (15d)	↓*Pseudomonas aeruginosa* infection impacts	Grass carp (*Ctenopharyngodon idella*)	Souza et al. (2019) [64]
	50–100 mg/kg fish feed (75d)	↑serum and splenic ADA, metabolites of NOx, MPO, CAT activities, resistance to *Pseudomonas aeruginosa* ↓NTPDase activity ↔growth performance	Grass carp (*Ctenopharyngodonidella*)	Baldissera et al. (2019) [65]
	200–300 mg/kg fish feed	↑ MDA, SOD, GPO, GSH, PC, RB, alternative pathway complement (ACP), Immune-related genes, Lys, β-2 M, CC3, IgM and SOD, GPO, Nrf2, and natural killer-cell enhancing factor (NKEF-β), TLR22, IL-1β, TNFα, hepcidin mRNA expression ↑resistance *vs Flavobacterium columnaris*	Rohu (*Labeo rohita*)	Harikrishnan et al. (2021) [66]
	50 mg/kg (28d)	↓TBARS, ALT, ALP, GGT ↑ Lys, MPO, GPO ↓presence of *Aeromonas*	Hybrid sturgeon (*Acipenser baeri* ♀ × *A. schrenckii* ♂)	Xu et al. (2021) [62]
Grape seed proanthocyanidins (GSPs)	250 mg/kg body mass intragastriced	↓pro-inflammatory cytokines (TNF-α, IL-6, IL-1β) ↓lipogenic miRNAs, miR-33, miR-122 ↓CHOL, TG, HDL ↑LDL ↑anti-inflammatory cytokines (IL-10)	Grass carp (*Ctenopharyngodonidella*)	Lu et al. (2020) [67]
	400 mg/kg (60d)	↓TC, TG, LDL-C, phenylalanine, tyrosine, tryptophan, valine, leucine, isoleucine biosynthesis ↑HDL-C, linoleic and arachidonic acid metabolism	American eel (*Anguilla rostrata*)	Wang et al. (2022) [68]
	200 mg/kg fish feed (49d)	↑body crude protein, Lys activity, albumin ↓body crude lipid, triglyceride, total cholesterol, ALT-AST activities	Nile tilapia (*Oreochromis niloticus*)	Zhai et al. (2018) [69]
	100–400 mg/kg fish feed (60d)	↓cortisol, glucose, Na+, lactate, ROS ↑SOD, CAT	Nile tilapia (*Oreochromis niloticus*)	Yang et al. (2023) [70]
	800 mg/kg fish feed (42d)	↓negative impacts of cadmium toxicity ↓protease activity and intestine antioxidant potential	Pearl gentian grouper (*Epinephelus fuscoguttatus* ♀ × *E. lanceolatus* ♂)	Jia et al. (2021) [71]
	200 mg/kg fish feed (50d)	↑hepatopancreas development ↓mortality ↔hepatic phenolic concentration	European seabass (*Dicentrarchus labrax*)	Magrone et al. (2019) [72]
Polyphenol-enriched feed	100–200 mg/kg fish feed	↑IFN-ɣ, spleen MMCs ↓spleen MØ	European seabass (*Dicentrarchus labrax*)	Magrone et al. (2016) [73]
	100–200 mg/kg fish feed (150d; 480d)	↑kidney MMCs, tyrosinase and peroxidase activity	European seabass (*Dicentrarchus labrax*)	Arciuli et al. (2017) [74]
Micro-encapsulated grape pomace extract (MGPE)	0.8% fish feed (90d)	↑antioxidant capacity, beneficial organisms ↓negative impacts of high soybean inclusion	Rainbow trout (*Onchorynchus mykiss*)	Pulgar et al. (2021) [61]

As mentioned before, tannins were considered anti-nutritional factors in fish inducing intestinal injury and reducing growth performances, also due to their poor palatability. By the way, some studies have been carried out essaying grape seed condensed tannins (CT) due to their beneficial effects at some levels. In Chen et al. (2022) CT were tested in four diets with various concentrations of inclusion in the Chinese seabass (*Lateolabrax maculatus*) [53]. The diet with the highest concentration of CT, but added with polyethylene glycol (PEG), reduced their adverse effects, because an alteration of the intestinal metabolomics was ascertained (increase in butyric, isovaleric and phenylacetic acids and decrease in isobutyric and valeric acids). This because PEG bound CT neutralizing their activity [53]. In the same species, the CT supplementation, ranging between 200 mg/kg and 400 mg/kg, lowered the levels of serum lipopolysaccharides (LPS), which are the main constituents of the membranes of Gram negative bacteria that activate Toll-like receptor 4 (TLR-4) and modulate the immune response, due to their anti-inflammatory effects. In addition, the genera *Cetobacterium* and *Aeromonas* decreased, due to the inhibitory effect of CT, while *Clostridum* and *Bravinema* increased. It was also confirmed that grape seed CT inhibited trypsin, and as dietary CT increased from 0 to 400 mg/kg, intestinal trypsin activity reduced, following a dose-dependent pattern and indicating that they may affect fish's capacity to digest protein. Fish gut Na+, K + ATPase activity may be inhibited too as a result of this reduced activity. In diets for *L. japonicus* juveniles, the introduction of CT did not change the activity of CK, as was seen in this study, despite the fact that CT was demonstrated to decrease food absorption. This suggested that CT, with supplementation up to 400 mg/kg, had no effect on energy metabolism and growth performances [54]**.**

In *Litopenaeus vannamei* shrimp, whose information are limited, a similar study was conducted including grape seed CT in the diet with higher dosages (from 0.5 g/kg up to 4 g/kg). Here, growth and nutrient digestibility resulted to be maintained, while the expression of antioxidant-related genes and intestinal histomorphology changed. In fact, the intermediate supplementation up-regulated superoxide dismutase (SOD), glutathione peroxidase (GPO) and Nrf-2 mRNA levels and gene expression. At the same time, intestinal villus of specimens receiving increasing supplementations of grape seed CT showed several degrees of deformation and histomorphology lesions (higher villus length and lower villus width) [55]. In the Chinese sea bass (*Lateolabrax maculatus*), the same authors tested the supplementation of grams/kg of grape seed CT with milligrams/kg of aflatoxin B1 (AFB1) in order to evaluate their efficacy in preventing damages caused by dietary AFB1 and possible correlation between their concentration. In fact, AFB1 is recognized as one of the most potent carcinogens, known to damage aquatic animals' development and immunity, as well as endanger the safety of food [56]. At the end, it showed that a supplement of 1 g/kg of grape seed CT effectively protected against AFB1 toxicity by lessening the harmful effects that dietary AFB1 had on fish. This work justified the CT use in aquaculture to protect fish against harm caused by AFB1 [5]. In conclusion, tannins used in the right concentration and formulation could be a booster for fish growth, gut and metabolic health.

The same author (Peng et al.) demonstrated also that the progressive inclusion of CT in diets for Japanese seabass (*Lateolabrax japonicus*) did not affect growth performances, body composition and liver histology. At the same time, intraperitoneal fat ratio, tumour necrosis factor and Interleukin-6 (IL-6) decreased, while serum antioxidant capacity (TAOC) and catalase (CAT) increased [57,58]. It was also demonstrated an activity of CT enhancing the resistance to copper sulphate stress test of *L. japonicus* juveniles, probably activating Nrf2 and decreasing the cumulative mortality rate [58].

By the way, other forms of grape seed were tested concerning their effectiveness on fish. For example, low dose supplementation of grape seed extract (GSE) (100 mg/kg) in rainbow trout (*Oncorhynchus mykiss)* induced an up-regulation of complement component 3 (C3), lysozyme (Lys), omDB-3, interferon gamma (IFN- γ), and TNF-α in different mucosal tissues, while higher dose (200 mg/kg) induced a prevalent up-regulation in fish skin, whose mucus showed bactericidal activity against *Yersinia ruckeri*. At the histological examination, skin resulted to show higher epidermis thickness, goblet cell density, and volume density in the GSE groups. In addition, the intestine of the GSE group showed higher villus height, goblet cell density and intraepithelial lymphocytes the number of goblet cells in the epithelium of gill filaments increased, showing a general modulation of the growth performance and mucosal immunity induced by the supplementation of GSE [59].

In rainbow trout, high percentages were supplemented (from 10 up to 50 g/kg), reporting an improvement in specific growth rate and condition index and biochemical markers such as glucose and lipid peroxidation product. It was observed an upregulation of the expression of antioxidant genes such as CAT, GPO-1, and glutathione S-transferase A [60].

In order to reduce possible negative alterations in the inflammatory and antioxidant response, grape pomace extract was supplemented as feed-additive, in the form of micro-encapsulated (MGPE) in *Oncorhynchus mykiss* diet including high rate of soybean meal. The fish gut microbiome analysis revealed a diet-dependent shift in the bacterial communities after 60 days of treatment with commercial high soybean meal diet with 1.2% maltodestrine with 0.8% of MGPE, as well as a time-dependent decline in microbial richness and also growth performance and antioxidant capacity increased [61].

Last, but not least, the effects of grape seed proanthocyanidins (GSPs) (50 and 100 mg/kg) were tested on hybrid sturgeon juveniles. It resulted that 50 mg/kg supplementation of GSPs modified the microbiota and reduced the presence of *Aeromonas*. At the same time, in serum, TBARS, alanine aminotransferase (ALT), alkaline phosphatase (ALP) and γ-glutamyl transpeptidase (GGT) decreased, while Lys, myeloperoxidase (MPO) and GPO increased, demonstrating an improved antioxidant activity. With the supplementation of 100 mg/kg, aspartate aminotransferase (AST) and lactate dehydrogenase (LDH) were raised while ALT and GGT decreased [62].

Changing the target organ studied, GSE have been tested to reduce the liver damages in *Oncorhynchus mykiss* fed diets including oxidized fish oil, confirmed by negative liver biochemistry parameters and histopathology. It was demonstrated that the 0.1% GSE supplementation ameliorated the adverse effects and reduced hepatocytes hydropic (vacuolar) degeneration and high hepatosomatic index (HSI). Significant ameliorative results were also found in liver biochemistry parameters compared to the oxidized group, such as superoxide dismutase (SOD), glutathione (GSH), malondialdehyde (MDA), ALT, AST andALP [63].

The efficacy of grape pomace meal (GPM) was investigated to reduce hepatic oxidative stress in grass carp (*Ctenopharyngodon idella*) experimentally infected by *P. aeruginosa*. By enhancing the enzymatic and non-enzymatic antioxidant defence system, dietary supplementation with 300 mg/kg GPM prevented *P. aeruginosa*-induced liver damage. This protective effect occurred through prevention of excessive ROS and metabolites of nitric oxide (NOx) production as well as via prevention of lipid damage [64]. In a similar trial, GPM supplementation was able to stop all changes brought on by experimental infection with *P. aeruginosa* (down-regulation of Nucleoside Triphosphate diphosphohydrolase-NTPDase, with an increase in adenosine deaminase-ADA, metabolites of NOx, MPO and CAT activities). Based on these metabolic changes, purinergic signalling contributed to the pro-inflammatory profile of immune lymphatic organs during *P. aeruginosa* infection and to disease pathophysiology. GPM feed supplementation controlled the inflammatory response by modifying serum and splenic purinergic transmission in response to the metabolic alterations induced by *P. aeruginosa*, so it may help alleviate immunological and inflammatory reactions brought on by grass carp's [65].

GPM diet supplementation was also tested at different concentrations (0,100,200,300 mg/kg) in rohu fish (*Labeo rohita*) experimentally exposed to infections with *Fusobacterium columnaris*. When diets were enriched with 100, 200 and 300 mg GPM, growth rate, haematology, and biochemical parameters significantly increased in both normal and challenged fish treated with 200 mg of GPM inclusion. In addition, the activities of antioxidants and innate-adaptive immune parameters, such as MDA, SOD, GPO, GSH, phagocytic (PC), respiratory burst (RB) increased. The expression of genes associated to immunity, antioxidants, and anti-inflammation were also markedly increased in the tissues of the head kidney in fish with diets enriched with 200 mg and 300 mg of GPM. The levels of all the examined parameters were consistently lower in the challenged fish fed without GPM. The findings showed that 200 mg GPM inclusion diet—both healthy and challenged fish—was sufficient to dramatically improve growth rates, antioxidant status, and immune protection, rather than an higher inclusion (300 mg of GPM) [66].

Grape seed procyanidin extract (GSPE) was reported to have hypolipidemic and potentially anti-inflammatory functions in the liver, mediated by the lipogenic genes miR-33 and miR-122, regulating the lipid metabolism and improving immunity. In fact, there was a reduction in the high fat diet-induced increase of serum cholesterol, triglycerides, and HDL, but increased LDL content. GSPE significantly lowered the expression of pro-inflammatory cytokines (TNFα, IL-6, and IL-1β) and enhanced the expression of the anti-inflammatory cytokine IL-10 [67].

In American eels (*Anguilla rostrata*), GSPE could elevate serum levels of HDL cholesterol and lower levels of total cholesterol, triglycerides, and low-density lipoprotein cholesterol. In addition to upregulating the metabolisms of linoleic acid and arachidonic acid, GSPE may also suppress the metabolisms of phenylalanine, tyrosine, and tryptophan biosynthesis as well as valine, leucine, and isoleucine biosynthesis [68].

GSPs demonstrated to be powerful in the protection from oxidative stress-induced by heavy metals in fish, significantly affecting the levels of MDA, GSH, and total antioxidant capacity (TAOC) and activities of SOD, CAT, and GPO. In fact, in tilapia (*Oreochromis niloticus*) juveniles, dietary cadmium (Cd) induced growth retardation and oxidative stress in hepatopancreas, an additional fish organ connected to the liver [69].

Another very recent paper tested different supplementations of GSPE (0,100,200,400 mg/kg) in Nile Tilapia (*Oreochromis niloticus*) juveniles, confirmed a decrease in serum stress-related parameters, such as cortisol, glucose, Na^+^ and lactate, and an increase of the TAOC, upregulating the SOD and CAT activities, that reduced the levels of ROS [70].

Differently, the same GSPs, tested in pearl gentian grouper (*Epinephelus fuscoguttatus* female x *Epinephelus lanceolatus* male) diet at 400 mg/kg and 800 mg/kg to alleviate the negative effects of dietary cadmium on growth performance and health status, the two GSPs supplementations were unable to reverse the negative effects of Cd stress, with the exception of calcium and phosphorus levels in the whole body composition [71]. A similar activity reported against the stress induced by heavy metals (copper sulphate) was cited before [58].

Polyphenols extracted from seeds of red grape (“Nero di Troia” cultivar) were also tested in sea bass (*Dicentrarchus labrax* L.) at different supplementations (100, 200 mg/kg) in order to evaluate the effects on hepatopancreas. This organ contains an exocrine portion, but until now its function has poorly been investigated [72]. Hepatopancreas area sizes were larger in fish that had been treated, also linking a reduction in mortality in farmed fish to an enlargement of the hepatopancreas caused by polyphenol treatment [72]. The effect of the same extract was also evaluated in intestine, spleen and kidney of the same sea bass species [73,74]. In the spleen of treated fish, there was an increase of IFN–***γ***, enhancing an adaptive immune response, and a reduction of groups of pigmented macrophages (MØ), with a contemporary increase in melanomacrophage centers (MMCs), that confirmed the evidence for a protective spleen response brought on by polyphenol-enriched diet. This because MMCs are a nodular cluster of MØ with heterogeneous inclusions, such as cell breakdown products, dedicated to the deconstruction of exogenous and endogenous antigens [73]. The same MMCs were found to have a stimulation of tyrosinase and peroxidase activity of the kidney of sea bass fed the same GSP phytocomplex rich in catechins and epigallocatechins. Again, as a manifestation of a powerful and protective adaptive immune response, an increase in melano-macrophage activity is associated with a stimulation of cytoprotective functions against antigenic stimulants and pathogens provided by GSP [74].

In conclusion, in almost every study very positive results have been highlighted in terms of antioxidant activity, immune response and beneficial influence in the gut health of fishes treated with the addition or replacement of grape by-products in various forms. The intermediate percentage of substitution/addition with grape by-products in the aquafeed very often gave the best positive effects on fish health status.

**2. Growth performances, feed conversion rate and somatic indices of fish fed with grape by-products**

In general, intermediate substitution/addition of grape by-products fits better than too low or high concentrations, resulting in improved growth performances, morphological, viscerosomatic and hepatosomatic indices (Table 2).

**Table 2:** Growth performances, feed conversion rate and somatic indices of fish fed with grape by-products.

Grape by-product	Most significant dose/(Experiment time, days)	Main findings	Fish species	References
Grape seed extract (GSE)	20–30 g/kg fish feed (56d)	↑growth performances (final length, weight, WG) ↓FCR ↑WBC, serum total protein, globulin, Lys and total antibodies activity, antibacterial activity vs *Aeromonas hydrophila*	Common carp (*Cyprinus carpio*)	Mehrinaki et al. (2021) [75]
	1.05% fish feed (90d)	↑growth performance ↓feed utilization	Rainbow trout (*Onchorynchus mykiss*)	Kesbiç et al. (2019) [78]
	5–30% feed + dried *Ulva lactuca*	↑survival, SOD activity, meal acceptance, feed intake	Greenlip abalone (*Haliotis laevigata*)	Lange et al. (2014) [86]
	80 mg/kg fish feed (60d)	↑growth performance, protease activity, triglycerides, albumin, Lys ↔hematological parameters	Tambaqui (*Colossoma macropopum*)	Morante et al. (2021) [87]
	250 ppm +1000 ppm of vitamin E (42d)	↑WG, SOD activity ↓TBARS	Whiteleg shrimp (*Litopenaeus vannamei*)	Chien et al. (2023) [88]
	12 g/kg fish feed (25d)	↑ growth performance, myostatin1	Senegalese sole (*Solea senegalensis*)	Xavier et al. (2020) [79]
	12 g/kg fish feed (25d)	↑ growth performance, after thermal stress prevented ↑protein carbonylation content and ↓antioxidant glutathione	Senegalese sole (*Solea senegalensis*)	Xavier et al. (2021) [80]
Grape pomace	15% fish feed (56d)	↑average daily WG, weights gain, SGR, morphological indices, K, VI and HSI ↓FCR	Common carp (*Cyprinus carpio*)	Mahmoodi et al. (2023) [77]
	18% fish feed (83d)	↔growth performances ↑dry matter apparent digestibility, FCR	Rainbow trout (*Onchorynchus mykiss*)	Peña et al. (2020) [81]
	200 mg/kg fish feed (40d)	↑WG, SGR, K ↑SOD, GPO ↓serum glucose, cholesterol, triglyceride	Common carp (*Cyprinus carpio*)	Mohammadi et al. (2021) [76]
Grape seed proanthocyanidins (GSPs)	200–400 mg/kg fish feed (49d)	↑growth performance ↓AST, ALT, triglycerides, total cholesterol ↑Lys, albumin	Nile tilapia (*Oreochromis niloticus*)	Zhai et al. (2014) [85]
	100–400 mg/kg fish feed (60d)	↑growth performance with 280 mg/kg fish feed; ↑ MyoG, GH, IGF-1, IGF-2 ↓Mstn1	Nile tilapia (*Oreochromis niloticus*)	Yang et al., 2023 [70]
	400 mg/kg fish feed (63d)	↑WG, SGR ↑antioxidant enzyme activities ↓FCR, ALT, ALP cholesterol, triglyceride	Gold fish (*Carassius aratus*)	Jahnbakhshi et al. (2023) [89]
	1000 mg/kg fish feed (60d)	↑growth performance, survival rates, antioxidant enzyme activities	Rainbow trout (*Onchorynchus mykiss*)	Arslan et al. (2018) [82]
Grape seed oil	50% of oil (60d)	↑growth performance, α-amylase activity, trypsin, total alkaline protease and lipase activities	Rainbow trout (*Onchorynchus mykiss*)	Zamani et al. (2021) [83]
	50% of oil (60d)	↑final weight, WG, fat and feed efficiency ↑pepsin and trypsin enzymes ↓FCR	Rainbow trout (*Onchorynchus mykiss*)	Zamani et al. (2018) [84]

In the common carp (*Cyprinus carpio*), high supplementation (up to 30 g/kg) of GSE was used, in order to evaluate its general effect and the resistance against *Aeromonas hydrophila*. It was found that fish fed with GSE had significantly higher final length, final weight and weight gain, while feed conversion rate (FCR) was lower. In addition to that, the treated groups with 20 and 30 g/kg of GSE showed higher white blood cells (WBC) count, serum total protein, globulin, lysozyme activity, total antibodies activities. Some of these biochemical parameters were found significantly changed also in mucus, where protease and antibacterial activity against *Aeromonas hydrophila* were the highest in fish fed with 30 g/kg of GSE; however, the mortality rate was higher in GSE group [75].

In the common carp, GSPE and grape pomace were used too. The supplementation of GSPE induced a reduction of serum glucose, cholesterol and triglyceride levels, whereas an incrementation of SOD and GPO activity was noted in the group supplemented with 200 mg/kg of GSPE. Even if the treatment exhibited no significant effect on body weight at the end of the experiment, the group with supplementation of 200 mg of GSPE showed a greater Weight Gain (WG), Specific Growth Rate (SGR), and Condition Factor (K). So GSPE could improve growth performances and serum biochemical parameters already at the lowest inclusion [76]. In the same cyprinid species, supplementation at 15% of grape pomace was confirmed to have significantly better results in terms of zootechnical performances. Average daily WG, total WG, SGR and morphological indices, such as K, viscerosomatic index (VI) and HSI were found to be significantly higher, while the FCR was favourable. So, high supplementation of grape pomace (150 g/kg) could become a positive ingredient in common carp diet [77].

GSPE was also tested in Nile tilapia (*Oreochromis niloticus*) and the optimum level of dietary GSPE required to grow and deposit muscle protein was proved to be around 280 mg/kg. The expression of genes related to growth (GH, IGF-1 and IGF-2) and hyperplasia (MyoG) was boosted by GSPE supplementation. Hyperplasia was also enhanced by the activation of the IGFs/PI3K/Akt/TOR/S6K1/4 EBP1 pathway, and at the same time a decrease of the gene expression Mstn1, which is a negative regulator of myofiber differentiation [70].

Even in carnivorous species, such as rainbow trout, grape by-products were tested in relation to their growth performances with positive results [78].

In the Senegalese sole postlarvae, grape seed and other natural antioxidants, as curcumin and green tea extracts, were tested. Grape seed significantly improved growth performances and, at the same time, modulated muscle development incrementing the expression of the gene myostatin 1 [79]. In contrast to what we have seen so far about the antioxidant properties of the grape seed, oxidative damage increased in soles reared in standard conditions. However, after a thermal stress condition, it was verified that grape seed, depending on the exposure time, reduced both the decline in the antioxidant glutathione and the rise in protein carbonylation concentration [80].

In rainbow trout fry, important supplementation of grape pomace (up to 60 and 80 g/kg) were used. However, no significant differences were observed in fish growth when dietary inclusion of grape pomace was up to 18%, but a higher digestibility and feed conversion efficiency were detected in fry fed the diet supplemented with 60 g/kg of grape pomace [81].

Grape seed oil, rich in beneficial properties, was tested in various percentages of substitution in juveniles of rainbow trout, in order to replace fish oil in standard fish meal and to detect ameliorative results in terms of growth performances, survival rates and antioxidant enzyme activities [82]. Two studies agreed that grape seed oil could substitute fish oil, not only in small quantity (mg/kg), but also up to 50% when the best performances were recorded and after which fish growth declined [83,84].

GSPs were tested at supplementations up to 800 mg/kg on fingerling of tilapia (*Oreochromis niloticus*). It was demonstrated that dietary GSPs of 200–400 mg/kg improved tilapia fingerlings' growth and body composition and blood biochemistry markers associated with health status. In fact, growth performances were significantly improved while serum blood parameters such as AST, ALT, triglyceride and total cholesterol lowered [85].

GSE was also added to diets for molluscs in order to reduce mortality that occurred during summer due to the high-water temperatures. In the greenlip abalone (*Haliotis laevigata Donovan*), GSE and *Ulva lactuca (L.)* were added to commercial diet (5% and 30%). Thanks to the richness in antioxidants and bioactive compounds of both compounds, the survival of abalone significantly ameliorated at high water temperature (26 °C), as well as the increase in feed intake and meal acceptance and SOD activity [86].

Other minor species reared were taken into account, always using GSE. In the omnivorous fish tambaqui (*Colossoma macropomum*), the greatest results of weight increase, SGR and K were obtained with an inclusion of crude grape extract at 80 g per kg diet. At this concentration, also the fish immunity had a boost, incrementing lysozyme activities and albumin levels [87].

A particular commercial grape extract (Nor-grape 80®) was added in concentrations of 250, 500, 750, 1000 ppm and compared to a commercial diet already containing 1000 ppm vitamin E (control diet) for the white shrimp (*Litopenaeus vannamei*). Even if aquafeeds have long included vitamin E as antioxidant, the results of the present study showed that 250 ppm of Nor-grape 80® was preferable to add in conjunction with 1000 ppm of vitamin E to obtain greatest weight increase, ideal SOD activity, and lowest TBARS levels. In fact, shrimp fed a diet supplemented with 250 ppm Nor-grape 80 gained weight at a rate that was about 2.5 times greater than shrimp fed a diet containing 1000 ppm vitamin E. In agreement with the studies previously discussed, the intermediate concentrations of grape extract (250 and 500 ppm) showed to achieve the best results. On the contrary, the highest concentrations of Nor-grape 80® showed reduced growth performance and lower antioxidant activity [88].

In conclusion, as a comparison for the most farmed fish species and the experimental model, GPE were included in experimental diets for the goldfish (*Carassius auratus*). In parallel with the results obtained in the other fish species, also here, the most balanced supplementation of 400 mg/kg incremented WG and SGR while feed conversion ratio decreased. Mucosal skin immunity ameliorated, reporting higher Lys activity and protein level; in serum, triglyceride, ALT and ALP decreased [89].

**3. Quality of fillets/flesh of fish fed with grape by-products and shelf-life**

During the rearing phase, fish quality traits and flesh composition are affected by fish feeding composition. At the same time, grape by-products could be used both as antioxidants and preservatives of products in order to prolongate shelf-life. In this paragraph, the effects of grape by-products are evaluated considering two approaches: “*ex-ante*” approach, because they can influence changes in the quality of flesh and fillets during the fish rearing phase (Table 3); “*ex-post*” approach, when they are used as food technology additives in order to elongate the fillets shelf-life or fish processed products (Table 4).

**Table 3:** Quality of fillets of fish fed with grape by-products.

Grape by-product	Most significant dose/(Experiment time, days)	Main findings	Fish species	References
Grape seed extract (GSE)	1.05% fish feed (90d)	↔moisture, fat, ash	Rainbow trout (*Onchorynchus mykiss*)	Kesbiç et al. (2019) [78]
Grape seed proanthocyanidins (GSPs)	200 mg/kg fish feed (40d)	↑fillet protein content ↔fat, ash, moisture	Common carp (*Cyprinus carpio*)	Mohammadi et al. (2021) [76]
	400 mg/kg fish feed (63d)	↑fillet protein content ↓lipid levels	Gold fish (*Carassius auratus*)	Jahnbakhshi et al. (2023) [89]
	100–400 mg/kg fish feed (60d)	↑muscular fiber diameter, myoglobin content, pH, color, tenderness, water holding capacity, ALA, EPA, n-3 PUFA	Nile tilapia (*Oreochromis niloticus*)	Yang et al. (2023) [70]
	3% fish feed (84d)	↑Σn3-PUFA, Σn6-PUFA, ratio hypocholesterolaemic/hypercholesterolaemic fatty acids ↓atherogenic and thrombogenic indeces	Common carp (*Cyprinus carpio*)	Zorlu et al. (2022) [91]
Grape seed oil	100–200 mg/kg fish feed (90d)	↓red (a *) and yellow (b *) indexes, fillet hardness, total lipid content, chewiness, saturated fatty acids, Atherogenic Index, malondialdehyde ↑polyunsaturated fatty acids content	European seabass (*Dicentrarchus labrax*)	Tarricone et al. (2023) [92]
	22% fish feed (57d)	↑fillets' oxidative stability ↓omega-3 fatty acids	Rainbow trout (*Onchorynchus mykiss*)	Baron et al. (2013) [93]
	0–100% of oil (60d)	↑crude protein ↓moisture ↑EPA ratio ↓DHA ratio	Rainbow trout (*Onchorynchus mykiss*)	Zamani et al. (2021) [83]
	1000 mg/kg fish feed (60d)	↑EPA ratio ↓DHA ratio	Rainbow trout (*Onchorynchus mykiss*)	Arslan et al. (2018) [82]
Grape pomace	10% fish feed (56d)	↑moisture, crude protein, carbohydrate content ↓crude lipid, energy	Common carp (*Cyprinus carpio*)	Mahmoodi et al. (2023) [77]

**Table 4:** Shelf-life of fish products treated with grape by-products.

Grape by-product	Time	Main findings	Fish product	References
Grape skin and grape seed extract (GSE)	4 ± 1 °C for different intervals	↑shelf-life period, bacterial lag phase ↓primary oxidation change in L*, a*, b* values	Atlantic salmon fillets (*Salmo salar*)	Simoes et al. (2019) [94]
GSE + carboxymethyl cellulose-based coating	4 °C for 20 days (5 days intervals)	↑organoleptic properties ↓TBARS, TVB-N, bacteria, lactic acid bacteria, *Pseudomonas* spp.	Rainbow trout fillets (*Onchorynchus mykiss*)	Raesi et al. (2015) [95]
GSE-carvacrol microcapsules + chitosan film	5 °C for 14 days	↑thickness, moisture content, a*, b*, opacity, water vapor, oxygen and carbon dioxide permeability ↓L*, water solubility, mesophilic and psychrophilic bacteria ↑shelf-life of 4–7 days	Refrigerated salmon (*Salmo salar*)	Alves et al. (2018) [96]
	4 ± 1 °C for 20 days	↑shelf-life of 6–8 days	Refrigerated red drum fillets (*Sciaenops ocellatus*)	Li et al. (2013) [97]
GSE	−18 ± 1 °C for 6 months	↓protein oxidation	Frozen mackerel mince (*Scomber scombrus*)	Özalp Özen et al. (2018) [100]
	−18 ± 2 °C for 6 months	↓lipid oxidation, trimethylamine (TMA), TVB-N ↑shelf-life of 60 days	Frozen Indian mackerel mince (*Rastrellinger kanagurta*)	Sofi et al. (2022) [101]
Oil-in-water nano emulsions based in grape oil	2 °C for 14 days	↓increase of pH, TVB-N, lipid oxidation and hydrolysis ↑shelf-life of 10–12 days	Chilled flathead mullet fillets (*Mugil cephalus*)	Ameur et al. (2022) [98]
Grape pomace flour (GPF)	−18 °C for 180 days (30 days intervals)	↓TBARS, lipid oxidation -Alteration of the colour and appearance ↔odor	Salmon burger (*Salmo salar*)	Cilli et al. (2019) [99]

A systematic review and meta-analysis [90] confirmed that grape pomace fortified meat, fish, dairy products and plant origin food thanks to the high levels of polyphenols and dietary fibres. Dietary fibres are mostly made up of insoluble fibres like cellulose and hemicelluloses and some of them form chemical bonds with phenolic compounds to produce antioxidant dietary fibres, increasing the pomace's capacity to scavenge free radicals. As consequence, the use of grape pomace had the most beneficial effects on the nutritional quality and oxidative stability of fortified goods, which are manifested as higher polyphenol and total dietary fiber contents. By the way, change of colour and undesirable modifications in texture were considered adverse effects of the use of this organic by-product [90].

Many studies described tests made on fish fillet, in order to show a clear improvement in quality through the dietary grape by-product supplementation. It was ascertained that fillet protein content resulted to be significantly higher than the fillet of fish fed without grape by-products [[76], [77], [78],^83^,^89^]. On the contrary, the percentage of total fillet lipid showed to be significantly lower, changing the type of fatty acids present [77,89]. For example, Σn3-PUFA and Σn6-PUFA values, as well as hypocholesterolaemic/hypercholesterolaemic fatty acids ratio increased [91].

For example, in Nile Tilapia (*Oreochromis niloticus*) with dietary GSPE supplementation, the flesh hardness, chewiness and gumminess significantly decreased, while α-linolenic acid **(**ALA), eicosapentaenoic acid **(**EPA), and n-3 PUFA increased, probably due to the antioxidant activity of the feed additive. At the same time, also the meat quality enhanced, improving muscle fiber diameter, myoglobin content, pH, colour, tenderness and the water-holding capacity [70].

In rainbow trout, *Vitis vinifera* grape seed oil supplementation with 250, 500 and 1000 mg/kg were essayed resulting in n6 fatty acids levels significantly higher in fish fillet of all experimental group in comparison to the control one. The group supplemented with 250 mg of grape seed oil per kg of feed showed the highest n6 levels, while, between the 500 and 1000 mg/kg feed groups, there was no change. In fish meat the most prevalent fatty acids were shown to be palmitic acid (C 16: 0), in the saturated fatty acids (SFA) group, and saturated docosahexaenoic acid (C 22: 6n-3) in the PUFA category. In comparison to the control, all groups' EPA ratios dramatically improved; docosahexaenoic acid (DHA) ratio was significantly higher in the control group, richer in fish oil [82].

A replacement of fish oil with grape seed oil was tested in different percentages (0, 25, 50, 75, 100%) in five experimental diets for rainbow trout. At the end of the trial, analysing fillets, maximum levels of n-6 series PUFAs were found in the fillet of fish fed with the maximum concentration of grape seed oil. In fact, PUFA levels rose significantly as grape seed oil levels increased, while on the contrary, the total amount of n-3 fatty acids gradually dropped, also decreasing the n-3/n-6 ratio [83].

A particular cultivar of *Vitis vinifera* (L., 1753) from Apulia (southern Italy), the Canosina grape, small and thick-skinned berries, was used to extract its polyphenol (GPE) and test in the feeding of European sea bass (*Dicentrarchus labrax* L.) juveniles. Two concentrations (100 and 200 mg/kg) were essayed with a reduction in fillet hardness, red and yellow indexes and preventing lipid oxidation. In fact, already the lowest concentration (100 mg/kg) ameliorated the total fatty acids profile, lowering their total quantity and presence of SFA. The antioxidant properties were enhanced by the increased presence of PUFA content and the lower presence of MDA [92].

Substitution of fish meal with plant protein concentrate and fish oil with vegetable oil, including grape seed, was studied both with “a priori” approach and “a posteriori” approach having evaluated the rainbow trout fillet composition and changes of its properties during 14 days ice storage. Although the fatty acid profile was not significantly changed by the addition of plant protein concentrate, the fish fillets' oxidative stability was marginally increased when compared to the control diet. The fatty acid composition of the fish muscle generally matched the fatty acid profile of the oil used; in fact, due to the reduction of fish oil, a decrease of omega-3 fatty acids was recorded in muscle [93]. This result was also ascertained using grape seed oil.

Concerning food technology and the implementation of the shelf-life of fish products, different coatings incorporating GSE were used in various substrates. In addition to that, it was really important to evaluate the effect of these antioxidant and antimicrobial substance and their effect in fish fillets colour because this is significant sensory quality that affects customer choice [94]. For example, carboxymethyl cellulose-based coating incorporated with GSE and the essential oil of *Zataria multiflora* Boiss., rich in monoterpene phenols (thymol, carvacrol), were tested in rainbow trout fillets during cold storage. This coating assured the best organoleptic properties of the fillet treated, as well as a reduction in TBARS, total volatile basic nitrogen (TVB-N) and bacteria, lactic acid bacteria and *Pseudomonas* spp. [95].

As a coating, chitosan, a type of fiber taken from the exoskeleton of insects and the shells of crustaceans, was used in combination with grape seed and carvacrol microcapsules to elongate the shelf-life of refrigerated fish products. GSE-carvacrol filmed with chitosan was utilized on salmon (*Salmo salar*) fillets; a higher thickness, moisture content, opacity, water vapor, oxygen and carbon dioxide permeability were showed. On the contrary, pH, lightness, mesophilic and psychrophilic bacteria lowered. As a consequence, refrigerated salmon elongates shelf-life 4–7 days thanks to the antibacterial function of these natural preservatives [96]. A similar study, with GSE combined with chitosan on refrigerated fillets of red drum (*Sciaenops ocellatus*) supported the same outcomings, extending the shelf-life of fish product by 6–8 days [97].

In chilled flathead mullet (*Mugil cephalus*) fillets, the effects of oil-in-water nanoemulsions with grape and cinnamon essential oils were evaluated in 14 days storage time at 2 °C. Good results outcome, both in the sensory assessment and the microbiological analyses, that confirmed a shelf-life extension of the filles from 10 to 12 days. This because it inhibited the increase of pH and TVB-N, and elongated lipid oxidation and hydrolysis [98].

GPM was tested at two different percentages in salmon burgers; as expected, TBARS significantly decreased, retarding the lipid oxidation of the frozen product. Sensorially, however, even if the odor was not affected by this supplementation, the colour changed; this was a physical quality that must be under control, because it can influence consumers’ choices [94,99]. However, in contrast with grape seed extract (GSE), pomegranate rind extract at a concentration of 100 ppm, resulted to lower significantly lipid oxidation, decreasing TBARS, protein oxidation and increasing sulphydryl contents, in mince of mackerel (*Scomber scombrus*) during a 6 months frozen storage [100].

A recent study concerning this topic reported that GSE was more effective than papaya extract for its antioxidant and antimicrobial properties in frozen storage quality of Indian mackerel (*Rastrellinger kanagurta*). This occurred because GSE possesses six times higher concentration of phenolic content than the papaya extract, hence GSE antioxidant and antimicrobial activity are more powerful. In fact, GSE extended the shelf-life of the fish product 60 days, twice as much as the papaya seed extract did (30 days) [101].

## 2 Conclusions

Nowadays, aquaculture is a field in great development and needs to become more sustainable day by day, facing out the aquaculture paradox (fishing ocean fish in order to produce fish meal for reared fish). The present review showed that agricultural wastes, as the grape by-products (grape seed and grape pomace), are a valid source for a new high-quality and resilient aquaculture [102]. Grape by-products demonstrate to be precious phytonutrients that could be added to fish feed in order to implement growing performance and fish health during rearing conditions. Their properties were highlighted to be useful also concerning food technology, due to their antioxidant and antimicrobial activities, that can improve the shelf-life of fish products. Further trials with different techniques could moderate and eliminate anti-nutritional factors and negative effects of grape by-products. Indeed, fish feed supplementation with grape-by products should be desirable because the properties that such a precious waste could provide are still largely unknown. All of this being part of the circular economy concept, where the aquaculture is linked to the agri-by-products in order to implement a sustainable strategy of fish production.

## Funding statement

This project is part of the PRIMA Programme supported by the 10.13039/501100000780European Union.

## Data availability statement

The data that support the findings of this study are openly available.

## CRediT authorship contribution statement

**Martina Quagliardi:** Writing – review & editing, Writing – original draft, Formal analysis, Conceptualization. **Emanuela Frapiccini:** Writing – review & editing, Visualization, Validation. **Mauro Marini:** Writing – review & editing, Validation, Supervision. **Monica Panfili:** Writing – original draft, Validation, Supervision. **Agnese Santanatoglia:** Methodology, Formal analysis. **Manuella Lesly Kouamo Nguefang:** Methodology, Formal analysis. **Alessandra Roncarati:** Writing – review & editing, Writing – original draft, Conceptualization. **Sauro Vittori:** Writing – review & editing, Writing – original draft, Validation, Supervision, Funding acquisition. **Germana Borsetta:** Writing – review & editing, Writing – original draft, Validation, Funding acquisition.

## Declaration of competing interest

The authors declare that they have no known competing financial interests or personal relationships that could have appeared to influence the work reported in this paper.
